# Magnetic Resonance Imaging of Atherosclerosis Using CD81-Targeted Microparticles of Iron Oxide in Mice

**DOI:** 10.1155/2015/758616

**Published:** 2015-07-21

**Authors:** Fei Yan, Wei Yang, Xiang Li, Hongmei Liu, Xiang Nan, Lisi Xie, Dongliang Zhou, Guoxi Xie, Junru Wu, Bensheng Qiu, Xin Liu, Hairong Zheng

**Affiliations:** ^1^The Third Affiliated Hospital of Southern Medical University, Guangzhou 510500, China; ^2^Paul C. Lauterbur Research Center for Biomedical Imaging, Institute of Biomedical and Health Engineering, Shenzhen Institutes of Advanced Technology, Chinese Academy of Sciences, Shenzhen 518055, China; ^3^Shenzhen Key Laboratory for Molecular Imaging, Shenzhen 518055, China; ^4^Department of Physics, University of Vermont, Burlington, VT 05405, USA

## Abstract

The goal of this study is to investigate the feasibility of using CD81- (Cluster of Differentiation 81 protein-) targeted microparticles of iron oxide (CD81-MPIO) for magnetic resonance imaging (MRI) of the murine atherosclerosis. CD81-MPIO and IgG- (Immunoglobulin G-) MPIO were prepared by covalently conjugating, respectively, with anti-CD81 monoclonal and IgG antibodies to the surface of the tosyl activated MPIO. The relevant binding capability of the MPIO was examined by incubating them with murine bEnd.3 cells stimulated with phenazine methosulfate (PMS) and its effect in shortening T2 relaxation time was also examined. MRI in apolipoprotein E-deficient mice was studied in vivo. Our results show that CD81-MPIO, but not IgG-MPIO, can bind to the PMS-stimulated bEnd.3 cells. The T2 relaxation time was significantly shortened for stimulated bEnd.3 cells when compared with IgG-MPIO. In vivo MRI in apolipoprotein E-deficient mice showed highly conspicuous areas of low signal after CD81-MPIO injection. Quantitative analysis of the area of CD81-MPIO contrast effects showed 8.96- and 6.98-fold increase in comparison with IgG-MPIO or plain MPIO, respectively (*P* < 0.01). Histological assay confirmed the expression of CD81 and CD81-MPIO binding onto atherosclerotic lesions. In conclusion, CD81-MPIO allows molecular assessment of murine atherosclerotic lesions by magnetic resonance imaging.

## 1. Introduction

Coronary artery disease arising from atherosclerosis is a leading cause of death, responsible for about 30% of all deaths worldwide, with more than 80% cases occurring in developing countries compared to developed countries [1, 2]. Atherosclerosis occurs through a slowly progressing lesion formation and luminal narrowing of arteries whereby lipids, inflammatory cells, and extracellular matrix accumulation in the subendothelial space (intima) lead to plaque formation. Upon plaque rupture and thrombosis, cardiovascular diseases such as acute coronary syndrome, myocardial infarction, and stroke appear. In clinical practice, the early identification and characterization of atherosclerotic lesions remain challenging. Early diagnosis via imaging techniques is especially desirable to refine diagnosis, guide intervention, and monitor response to therapies [3].

Magnetic resonance imaging (MRI) has demonstrated substantial potential in phenotyping vascular disease. It has been achievable to characterize the vessel wall in atherosclerosis at a submillimeter level by taking advantage of inherent physicochemical properties. More importantly, magnetic resonance molecular imaging presents opportunities to image directly the biological processes of vascular disease at the molecular and cellular levels [4, 5]. By specifically targeting molecules differentially expressed in disease states, purpose-built molecular imaging probes can contribute to the molecular understanding of a range of diseases and potentially offer a means of clinical diagnosis [6].

CD81 (TAPA-1), a ubiquitously expressed tetraspanin protein, participates in diverse biological activities including hepatitis C virus (HCV) infection [7], plasma cell dyscrasias [8], B lymphocyte function [9], cell proliferation [10], differentiation [11], and cell migration [12]. Recent evidence suggests that endothelial CD81 is specifically upregulated in the endothelium of atherosclerotic plaques in human arteries [13]. These data indicate that endothelial CD81 can be used as a marker of early human atherosclerotic plaques. In this context, by developing a contrast agent targeted to this specific biomarker, it will be possible to provide us with insights into molecular understanding of atherosclerosis and to potentially offer a tool of clinical diagnosis.

Micron-sized particles of iron oxide (MPIO) have been used for cellular imaging and tracking for a long time [14]. MPIO provide excellent contrast effect for magnetic resonance imaging, through delivering a high payload of iron oxide [15]. The conjugation of antibodies to MPIO further confers target specificity and binding affinity and provides a way of molecular detection of vascular pathologies. In this study, we report an antibody-conjugated MPIO probe for targeted MRI and applied it for detection of atherosclerotic lesions of apolipoprotein E-knockout (apoE−/−) mice.

## 2. Materials and Methods

### 2.1. Materials

MPIO with 4.5 *μ*m diameter were purchased from Invitrogen (Carlsbad, CA, USA). Monoclonal rat anti-mouse CD81 antibody, FITC-labeled anti-CD81 antibody, and IgG-1 antibody were purchased from BD Biosciences (San Jose, CA, USA). PMS, DiI, and 4′,6-diamidino-2-phenylindole (DAPI) were obtained from Sigma-Aldrich (St. Louis, MO, USA). All other reagents were of analytical grade. Mouse brain microvascular endothelial cells (bEnd.3) were purchased from the American Type Culture Collection. Female apoE−/− mice, weighing about 20 g (8 weeks old), were obtained from Beijing Weitong Lihua Test Animal Co. (Beijing, China).

### 2.2. Antibody Conjugation to Iron Oxide Microparticles

MPIO with p-toluenesulfonyl (tosyl) reactive surface groups were used to covalently conjugate with anti-CD81 monoclonal antibodies and IgG-1 control antibodies according to the previous report [16]. In brief, 30 *μ*g of purified monoclonal rat anti-mouse antibodies for CD81 or IgG-1 was covalently reacted with 6 × 10^7^ MPIO, by incubation at 37°C for 20 hours. The resulting antibody-conjugated MPIO were then washed twice in phosphate buffered saline (PBS) containing 0.1% bovine serum albumin (BSA) at 4°C. To block remaining active tosyl sites, these MPIO were further incubated with Tris buffer (0.1 mol/L, 0.1% BSA, pH 7.4) for 4 hours at 37°C, followed by rinse in PBS (0.1% BSA) at 4°C for 5 minutes, and stored at 4°C until use.

### 2.3. Characterization of CD81-Targeted UCA

50 *μ*L of FITC-labeled targeted MPIO suspension (1 × 10^8^ particles/mL) was applied to the microscope slide. A cover slip was used to cover the sample. Morphologic characteristics of targeted MPIO were determined under a fluorescent microscope (Olympus, Tokyo, Japan). The amount of the antibody conjugated to MPIO was determined through detecting the decrease of fluorescence intensity of FITC-labeled CD81 antibodies before incubation and after incubation with MPIOs.

### 2.4. In Vitro Binding of CD81-MPIO to PMS-Stimulated bEND-3 Endothelial Cells

The murine bEnd.3 endothelial cells (1 × 10^5^ cells/well) were cultured in plates as monolayer in Dulbecco's Modified Eagle's Medium (DMEM), supplemented with 10% fetal calf serum and 1% penicillin-streptomycin solution (1% v/v penicillin-streptomycin solution containing 5,000 units penicillin and 5 mg streptomycin per mL), and maintained in a humidified atmosphere containing 5% CO_2_ at 37°C. Cells were seeded in 6-well plates overnight to allow cell adhesion. 5 *μ*M of PMS was added to the media and further incubated for 16 h to induce expression of CD81 according to our previous report [17]. Stimulated cells were incubated in duplicate with various concentrations of CD81-MPIO or control IgG-MPIO (1 × 10^5^, 1 × 10^6^, or 1 × 10^7^ per mL medium) for 30 minutes at room temperature. Unbound MPIO were removed by extensive washing with PBS. MPIO binding to cells was assessed in 4 fields of view by light microscopy (Leica DM R; ×20 objective). Each group was repeated three times to obtain the quantitative data. FITC-labeled anti-CD81 antibody-conjugated MPIO (1 × 10^7^ particles per mL medium) were also used for incubation with PMS-stimulated cells. After rinse, these cells were fixed with 80% ethanol, stained with DiI and DAPI dyes, and examined under fluorescent confocal microscopy (Leica TCS SP5, Wetzlar, Germany).

### 2.5. In Vitro MRI of Cell Phantoms

Stimulated cells were incubated with various concentrations of CD81-MPIO or control IgG-MPIO (1 × 10^5^, 1 × 10^6^, or 1 × 10^7^ particles per mL medium). The incubated cells were rinsed, resuspended, and embedded in 2% high grade agarose. MRI was performed using a 3T clinical MR scanner with a small animal coil. T2-weighted MR images were acquired using the following parameters: multicontrast-spin echo (se-mc) sequence, TR = 5000 ms, TE from 10.6 to 53 ms, FOV = 25 × 62 mm, and slice thickness = 1.0 mm. The signal intensity of the T2-weighted MRI was used to calculate the T2 value of each sample through an analysis program provided by the MRI scanner.

### 2.6. ApoE−/− Mice and Left Ventricular Injection of MPIO

All animal experiments were performed in compliance with the relevant laws and institutional guidelines. Homozygous apoE−/− mice, bred in a pathogen-free room, with constant temperature and humidity, were weaned at 3 weeks of age and transferred to Western diet (21% milk fat, 0.15% cholesterol) for 32 weeks. ApoE−/− mice were terminally anesthetized by isofluorane inhalation. The chest cavity was exposed while the heart was beating. Mice were injected via the left ventricle with CD81-MPIO (*n* = 5 mice), control IgG-1-MPIO (*n* = 5 mice), or plain MPIO (*n* = 4 mice). 1 × 10^7^ MPIO in 100 *μ*L PBS (about 3 mg iron/kg body weight) was administrated for each mouse and allowed to circulate for 30 minutes. Before and after injection of MPIO, the mice were imaged.

### 2.7. In Vivo MRI Analysis

In vivo MRI was performed at 7.0 Tesla using a 35 mm birdcage coil and mouse cradle. Animals were initially anesthetized with a 4% isoflurane/air gas mixture delivered through a nose cone and maintained under anesthesia with a 1.5–2% isoflurane/air gas mixture. Cardiac and respiratory signals were continuously monitored using an in-house developed ECG- and respiratory gating device. The following MRI sequences were used: Fast Low Angle Shot (FLASH): TR = 30.7 ms, TE = 1.8 ms, slice thickness = 0.93 mm, slices = 4, interslice = 0.2 mm, Flip angle = 18°, and matrix = 256 × 256. The total imaging time for each time-point was less than 40 min. Aortic root low signal areas were measured by drawing an area of interest around the outer wall of the aortic root using ImagePro Plus version 6.1. The threshold of low signal, corresponding to MPIO binding, was defined to be 3 standard deviations below the mean signal intensity of the precontrast root on signal intensity histograms. This threshold was applied to sequential MR slices through the aortic root, before and after MPIO contrast, to determine the mean area of MPIO low signal contrast. The contrast-to-noise ratios (CNR) of identified MPIO-positive lesion areas were compared to the CNR of equivalent areas on the precontrast images as CNR = blood pool signal − lesion signal/standard deviation of the noise as previously described [16, 18].

### 2.8. Tissue Harvesting and Histology

After performing in vivo MRI, animals were euthanized and perfused in situ with PBS and then 4% PFA via the left ventricle. Mouse aortas were dissected for histological phenotyping, covered with Tissue-Tek (Sakura), and then frozen in liquid nitrogen vapor. Aortas were cut with a cryostat microtome (CM1950; Leica, Heidelberg, Germany). Each section was 6 *μ*m thick. Immune-staining was done using 1 *μ*L Armenian hamster anti-mouse CD81 antibody (0.5 mg/mL, 1 : 50 dilution in PBS; BD Biosciences) and 1 *μ*L anti-Armenian hamster IgG secondary antibody (0.1 mg/mL 1 : 100 dilution in PBS; BD Biosciences). Cell nuclei were counterstained with hematoxylin. Tissue sections were viewed under an optical microscope (Olympus, Tokyo, Japan).

### 2.9. Statistical Analysis

All data are expressed as mean ± SEM. All multiple comparisons were made by one-way analysis of variance (ANOVA). All statistical tests were performed using SPSS for windows (Version 19.0; SPSS). *P* < 0.05 and *P* < 0.01 were considered to show significant and highly significant differences, respectively.

## 3. Results

### 3.1. Characterization of the CD81-MPIO

**Figure 1:**
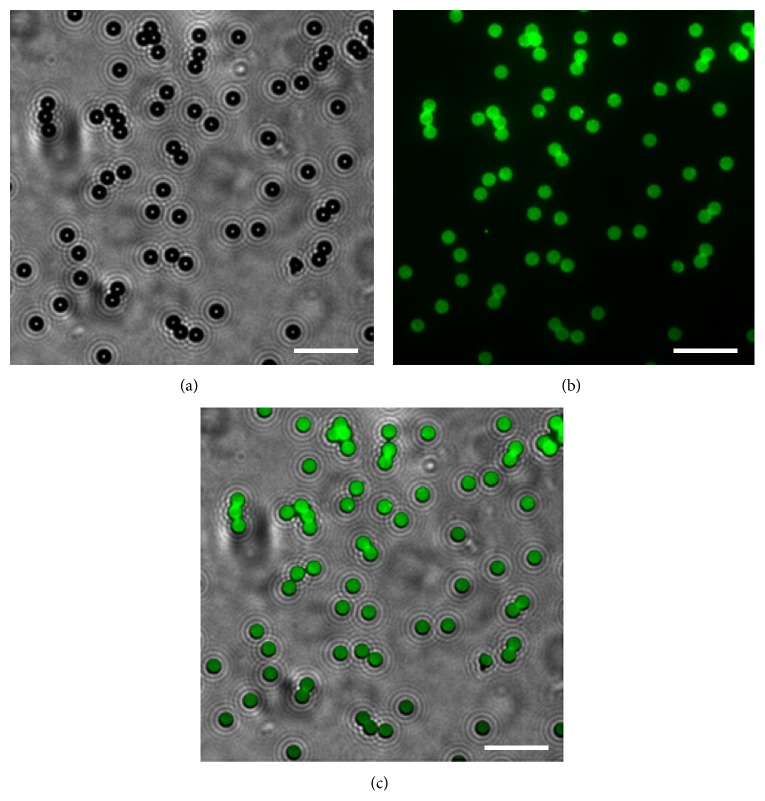
Characterization of the CD81-targeted MPIO (CD81-MPIO). (a) Bright micrograph of FITC-labeled CD81-targeted MPIO. (b) Fluorescent micrograph of FITC-labeled CD81-targeted UCA. (c) Merged image from (a) and (b) indicates binding of anti-CD81 antibodies to MPIO (bar = 20 *μ*m).

To test whether antibodies can bind to MPIO, the particles were incubated with FITC-labeled anti-CD81 antibodies.  Figure 1(a) from bright field image shows CD81-MPIO are dispersive spherical particles. Under fluorescent microscope, the surfaces of the fluorescent-labeled CD81-MPIO appear green (Figure 1(b)). The merged image showed green areas may well coincide with the surfaces of these particles, which indicates the successful conjugation of anti-CD81 antibodies to MPIO (Figure 1(c)). Incubation of MPIO with excess CD81 antibodies resulted in the conjugation of 1.5 × 10^5^ antibody molecules per MPIO.

### 3.2. In Vitro Binding of CD81-MPIO to PMS-Stimulated bEND-3 Endothelial Cells

**Figure 2:**
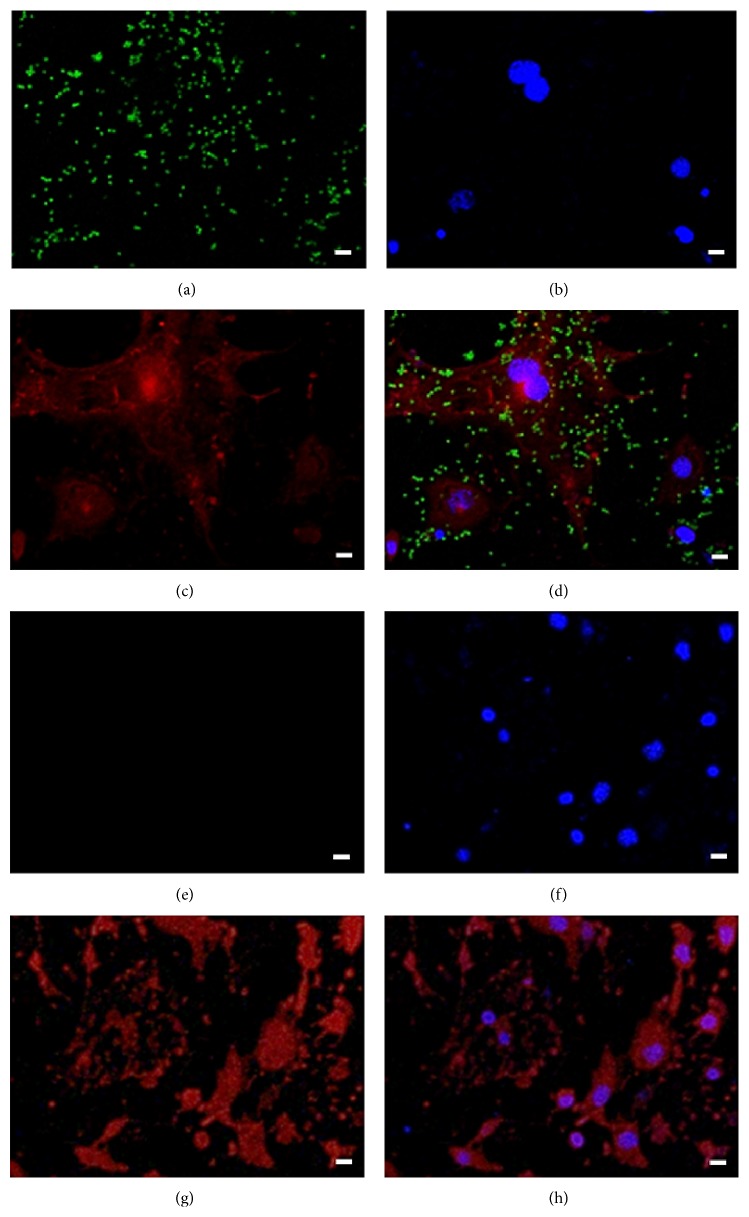
Confocal laser scanning microscopy shows CD81-MPIO binding to bEnd.3 cells. The cells were stimulated with 5 *μ*M PMS for 16 h and incubated with FITC-labeled CD81-MPIO or IgG-MPIO. After that, the cells were stained by DAPI and DiI dyes and examined under confocal laser scanning microscopy. (a) The MPIO particles appeared green. (b) The nuclei showed blue. (c) The cell membrane was stained red. (d) The merged image from (a), (b), and (c) showed CD81-MPIO can bind to PMS-stimulated cells. (e–h) As a control, IgG-MPIO did not bind to the PMS-stimulated bEnd.3 cells (bar = 20 *μ*m).

**Figure 3:**
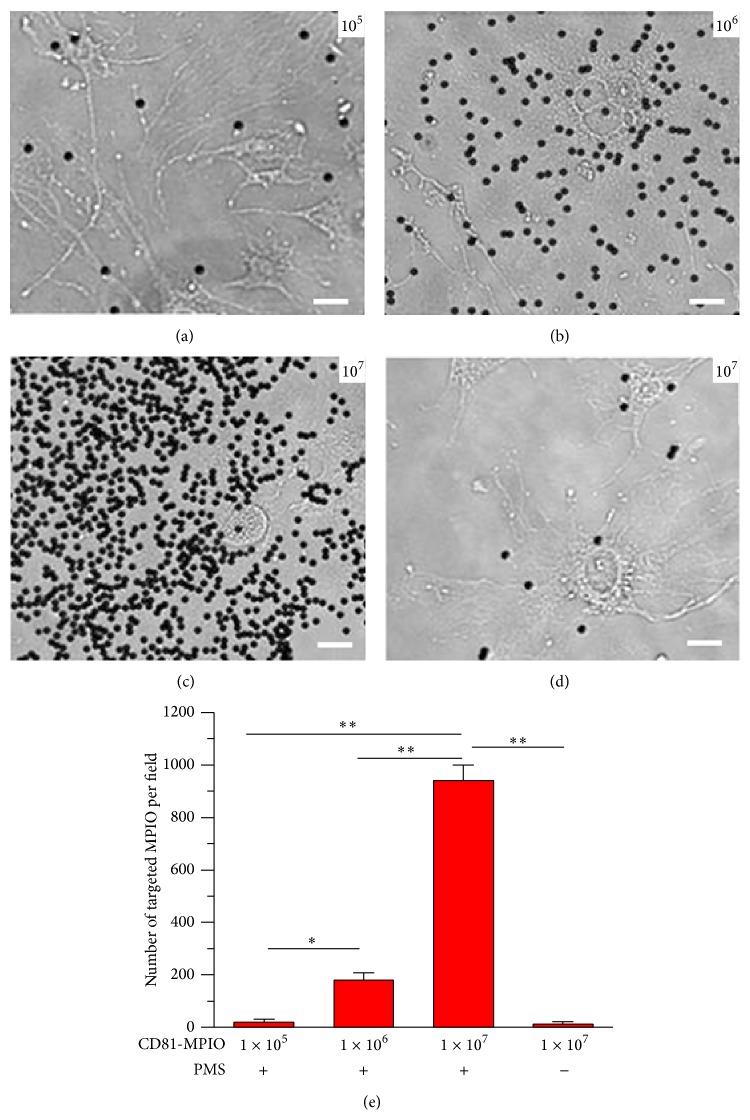
Concentration dependence of CD81-MPIO binding. Various concentrations of CD81-MPIO were used for examining binding efficiency. 1 × 10^5^ (a), 1 × 10^6^ (b), and 1 × 10^7^ (c) CD81-MPIO particles were used for examining binding efficiency to the PMS-stimulated bEnd.3 cells. (d) 1 × 10^7^ CD81-MPIO was used for the nonstimulated bEnd.3 cells. (e) Quantitative assay of the number of MPIO adhered onto bEnd.3 cells from six random view fields (bar = 20 *μ*m).

Rohlena et al. (2009) reported that CD81 proteins can be induced by phenazine methosulfate (PMS) and resulted in upregulation of CD81 expression. To examine binding affinity, FITC-labeled CD81-MPIO or FITC-labeled IgG-MPIO were incubated with PMS-induced bEnd.3 cells. Results showed a large number of CD81-MPIO bound to the surface of stimulated bEnd.3 cells (Figures 2(a)–2(d)). By contrast, IgG-MPIO did not bind to the surface of stimulated bEnd.3 cells (Figures 2(e)–2(h)). With the increase of MPIO concentration, the number of adhesive CD81-MPIO was gradually improved (Figures 3(a)–3(c)). Otherwise, just a small number of CD81-MPIO bound to the surface of nonstimulated bEnd.3 cells (Figure 3(d)). Quantitatively, there were only 18.26 ± 13.11 MPIO bound to the PMS-stimulated cells when 1 × 10^5^ CD81-MPIO particles were used, while 180 ± 28, 940 ± 59 MPIO bound to these cells when using 1 × 10^6^, 1 × 10^7^ CD81-MPIO particles, respectively (*P* < 0.01) (Figure 3(e)). In contrast, there were just 12 ± 9 particles bound to nonstimulated bEnd.3 cells even if 1 × 10^7^ CD81-MPIO particles were used.

### 3.3. In Vitro MRI of Cell Phantoms

**Figure 4:**
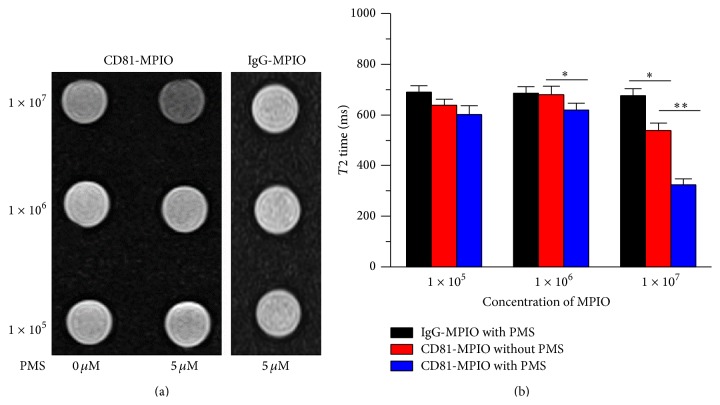
In vitro MR images of CD81-MPIO. (a) T2-weighted imaging of the control cells and PMS-stimulated cells incubated with various concentrations of CD81-MPIO (left panel) or IgG-MPIO (right panel) at 1 × 10^5^, 1 × 10^6^, or 1 × 10^7^ particles. (b) The T2 relaxation time of the control cells and the PMS-stimulated after incubation with CD81-MPIO or IgG-MPIO.

In order to examine whether CD81-MPIO were able to improve contrast effects of endothelial cells, stress-stimulated or non-stress-stimulated bEnd.3 cells were incubated with various concentrations of CD81-MPIO or IgG-MPIO and imaged by MR imaging system.  Figure 4(a) demonstrated that signal intensity of the cells received with CD81-MPIO in the T2-weighted images significantly decreased when compared with non-stress-stimulated cells received with CD81-MPIO or stress-stimulated cells received with IgG-MPIO. The quantitative signal intensities of the images by calculating the transverse proton relaxation time (T2) were shown in Figure 4(b). The mean signal intensities ranged between 323.86 ± 23.2 and 549.8 ± 26.1 for PMS-stimulated cells, significantly lower than that of nonstimulated cells with 537.6 ± 30.1 and 650.5 ± 32.3 (*P* < 0.01 for 1 × 10^7^ CD81-MPIO, *P* < 0.05 for 1 × 10^6^ CD81-MPIO). There is no significant difference in mean signal intensities when 1 × 10^5^ targeted MPIO were used (*P* > 0.05). Also, there had not been significantly change of the T2 relaxation time for the cells received with IgG-MPIO.

### 3.4. In Vivo MRI Analysis

**Figure 5:**
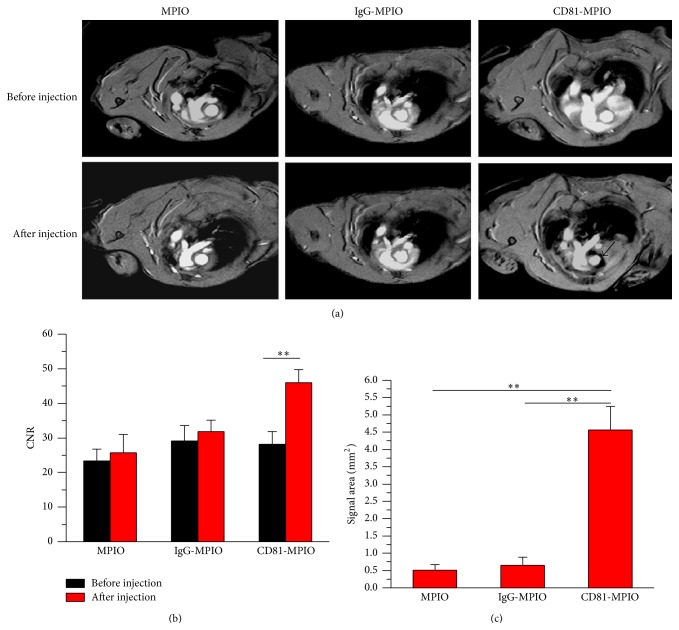
In vivo MR images of aortic root after MPIO injection (a) Representative MR images of the aortic root in apoE−/− mice before or after injection with MPIO (left), IgG-MPIO (middle), or CD81-MPIO (right). The arrow points out the low signal areas after CD81-MPIO injection. (b) Contrast-to-noise ratio (CNR) of MPIO-positive lesion areas was significantly increased (*P* < 0.05 at 14 weeks; *P* < 0.01 at 20 and 30 weeks) after injection of CD81-MPIO compared to equivalent lesion areas on precontrast images (mean ± SD), with no significant difference in post-MPIO CNR between time-points. Scale bars = 1 mm. (c) Quantitative analysis of MRI data. Mean area (±SD) of low MR signal areas in aortic roots at baseline and 30 and 60 minutes after injection of PV-MPIO.

To further determine whether CD81-MPIO can detect atherosclerotic lesion in vivo, MRI was performed before and after injection of either CD81-MPIO, negative control IgG-MPIO, or plain MPIO. Representative MR images of aortic root atherosclerotic lesions are shown in Figure 5(a). MR images showed highly conspicuous areas of low signal after CD81-MPIO injection, with minimal MR signal effects observed in precontrast images and in aortic root images of mice injected with control IgG-MPIO or plain MPIO. The contrast-to-noise ratios (CNR) of MPIO-positive lesion areas and adjacent blood pool increased by 63.01% after CD81-MPIO injection, compared to precontrast aortic lesion areas (*P* < 0.01). No significant differences in lesion CNR were observed for IgG-MPIO or plain MPIO between time-points (*P* > 0.05) (Figure 5(b)). Quantitative analysis of the area of CD81-MPIO contrast effects in the aortic roots showed 8.96- and 6.98-fold increase in the areas of low MR contrast effects compared to IgG-MPIO or plain MPIO (*P* < 0.01) (Figure 5(c)).

### 3.5. H&E Staining and Immunohistochemical Assay

**Figure 6:**
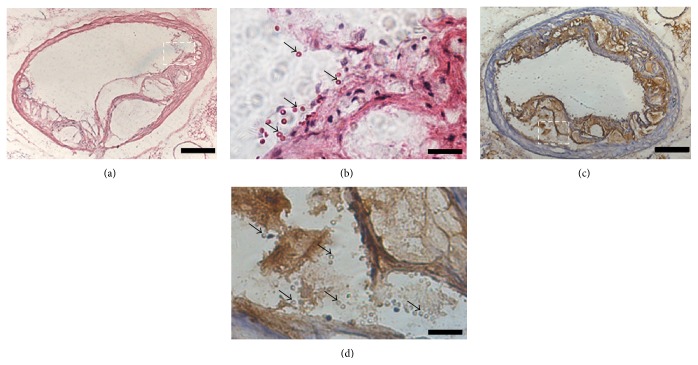
Histological identification of MPIO. (a) Representative H&E staining of an aortic artery section from the mice receiving CD81-MPIO. (b) Enlargement of the area from (a) shows MPIO on the surface of the atherosclerotic plaque (arrows). (c) Immunohistochemical analysis CD81 expression in atherosclerotic plaque associated with the aortic root. 6 *μ*m contiguous histological sections were taken in a similar orientation to the ex vivo MRI of the aortic root. Immunohistochemical staining was used to demonstrate CD81 expression (brown). (d) Enlargement of the area from (c) shows CD81-positive cells inside the atherosclerotic plaque. Scale bars are 100 *μ*m for images (a) and (c), 20 *μ*m for images (b) and (d).

Histological assessment of CD81-MPIO attachment and immunohistochemical assessment of CD81 expression were performed within the atherosclerotic lesions in the aortic root. H&E staining showed the binding of the targeted MPIO was confined to regions of the root affected by atherosclerosis but not to lesion-free areas (Figures 6(a) and 6(b), arrow). Immunohistochemical staining showed significantly high levels of CD81 expression in the atherosclerotic lesions in the aortic root (Figures 6(c) and 6(d), brown).

## 4. Discussion

Atherosclerosis is a complex, chronic disorder involving inflammatory and proliferative signaling pathways. The vascular endothelium is the principal regulatory interface that governs the recruitment of mononuclear cells to sites of injury and inflammation through the upregulation of various adhesion molecules such as VCAM-1, ICAM-1, and P-selectin [19, 20]. Therefore, exploring specific molecular markers which are suitable for constructing imaging probes for diagnosis is of importance. Previous studies have showed that endothelial CD81 is an ideal molecular marker for evaluating the atherosclerosis prior to the full-blown inflammatory response [13]. Here, we demonstrated the MR molecular imaging of atherosclerotic lesion in apoE−/− mice using CD81-targeted MPIO (Figure 5).

In MR molecular imaging field, a number of strategies have been employed; for example, gadolinium based contrast agents are used for detecting biological activity related to lesions. However, the relaxivity effects achievable are relatively modest. In comparison to Gd, iron oxide agents including ultrasmall superparamagnetic particles of iron oxide (USPIO) (20–50 nm diameter), superparamagnetic particles of iron oxide (SPIO) (60 to approximately 250 nm), and micrometer-sized particles of iron oxide (MPIO) (0.9–8 *μ*m) are superior in improvement of MR contrast. They create hypointense areas and result in a black appearance on the MR image on T2- and T2^*∗*^-weighted MR images. For molecular imaging of endovascular targets, MPIO offer a number of important advantages. First, the relatively large size of MPIO makes them less susceptible to nonspecific vascular egress or uptake by endothelial cells than nanoparticles; thus they can retain specificity for endothelial molecular targets [21]. Secondly, unbound MPIO have been shown to clear rapidly from the blood stream, which minimize the background blood phase contrast [22]. Conversely, USPIO may cause high background contrast due to its long blood half-life (up to 24 h), making it difficult to distinguish specific contrast effects from normal tissue heterogeneity and other susceptibility artifacts. Thirdly, taking advantage of the contrast “blooming effect” of MPIO, a small number of MPIO can create potent hypointense contrast on T2^*∗*^-weighted images, thereby greatly enhancing contrast-sensitivity. It is especially valuable for low-abundance endothelial molecular targets. In particular, MPIO convey a payload of iron oxide (typically 0.1–1.6 pg iron/MPIO particle), which is orders of magnitude greater than that contained in nanometer-sized particles [23]. In fact, ligand-conjugated MPIO targeting endothelial-expressed markers such as vascular cell adhesion molecule-1 (VCAM-1) [24], intercellular adhesion molecule-1 (ICAM-1) [25], platelet endothelial cell adhesion molecule-1 (PECAM-1) [26], GP IIb/IIIa [27], and P- and E-selectin [16, 28] have been developed and used to make disease pathogenesis such as inflammation, atherosclerosis, and angiogenesis “visualized.”

In this work, we constructed CD81-MPIO by conjugating anti-CD81 antibodies with MPIO. The resulting targeted MPIO show a highly effective binding with PMS-stimulated bEnd.3 cells (Figure 2), which resulted in significantly lower signal intensity in the T2-weighted images compared with the control nonstimulated cells (Figure 3). These data indicated that CD81-MPIO may be an excellent MR molecular imaging probe to detect the expression of CD81 proteins. It is worthy to point out that we imaged the aortic root in its short axis by using CD81-MPIO, which is in common with those earlier studies [18]. The main reasons can be summarized by the following facts: (1) atherosclerotic lesions develop early in the aortic root of apoE−/− mice [29, 30]; (2) by taking advantage of the aortic valve's position in relation to the chambers of the heart, the aligning of both the imaging plane and circumferential orientation can be easily provided.

As shown in Figure 5, we observed highly conspicuous areas of low signal following the CD81-MPIO injection. By contrast, we did not observe any change in mice receiving control-MPIO. In addition, the distribution of CD81-MPIO was spatially related to the areas of the root showing atherosclerotic lesions which have a high-level expression of CD81. This agrees with the report that CD81 is most strongly upregulated in vascular endothelium overlying plaque [13]. It is still to point out that the interest of our work is to design a novel probe to characterize the CD81 expression in plaque for future possible application to prevent complications induced by plaques: rupture and thrombosis. Although further investigations are still needed, our study provided an alternative tool to explore the possible changes of CD81 protein from the nascent to the mature and complicated plaque.

## 5. Conclusions

This study demonstrated the successful preparation of the CD81-MPIO. Our initial experience has shown its usefulness for in vivo molecular MRI to detect atherosclerotic lesion. More importantly, MR imaging using CD81-MPIO as a contrast agent may provide a noninvasive tool to explore expression of CD81 and its corresponding gene functions linked to the genesis, development, prognosis of atherosclerosis, and susceptibility to antiatherosclerosis drugs.
