# Erratum to “Magnetic Resonance Imaging of Atherosclerosis Using CD81-Targeted Microparticles of Iron Oxide in Mice”

**DOI:** 10.1155/2018/8093438

**Published:** 2018-10-18

**Authors:** Fei Yan, Wei Yang, Xiang Li, Hongmei Liu, Xiang Nan, Lisi Xie, Dongliang Zhou, Guoxi Xie, Junru Wu, Bensheng Qiu, Xin Liu, Hairong Zheng

**Affiliations:** ^1^The Third Affiliated Hospital of Southern Medical University, Guangzhou 510500, China; ^2^Paul C. Lauterbur Research Center for Biomedical Imaging, Institute of Biomedical and Health Engineering, Shenzhen Institutes of Advanced Technology, Chinese Academy of Sciences, Shenzhen 518055, China; ^3^Shenzhen Key Laboratory for Molecular Imaging, Shenzhen 518055, China; ^4^Department of Physics, University of Vermont, Burlington, VT 05405, USA

In the article titled “Magnetic Resonance Imaging of Atherosclerosis Using CD81-Targeted Microparticles of Iron Oxide in Mice” [1], the images in the IgG-MPIO column in Figure 5 did not clearly show the difference between the movement before and after injection. Therefore, they have been replaced with a locally enlarged higher-resolution version in place.

In addition, there was an error in the legend of Figure 5 where “(*P* < 0.05 at 14 weeks; *P* < 0.01 at 20 and 30 weeks)”, “(mean ± SD), with no significant difference in post-MPIO CNR between time-points. Scale bars = 1mm”, and “at baseline and 30 and 60 minutes after injection of PV-MPIO” should be removed. The corrected Figure and legend are as follows.

## Figures and Tables

**Figure 5:**
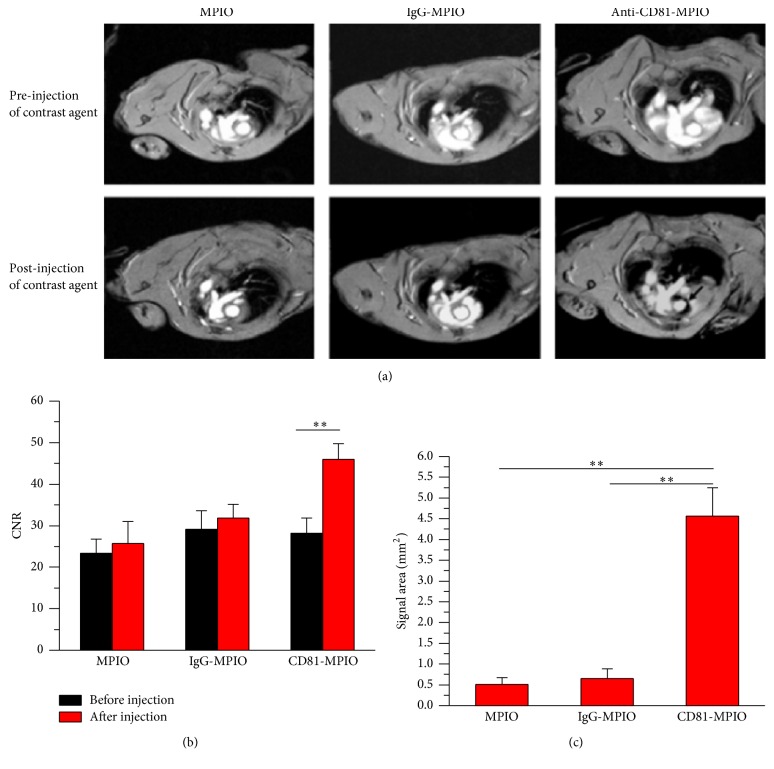
In vivo MR images of aortic root after MPIO injection.(a) Representative MR images of the aortic root in apoE−/− mice before or after injection with MPIO (left), IgG-MPIO (middle), or CD81-MPIO (right). The arrow points out the low signal areas after CD81MPIO injection. (b) Contrast-to-noise ratio (CNR) of MPIO-positive lesion areas was significantly increased after injection of CD81-MPIO compared to equivalent lesion areas on precontrast images. (c) Quantitative analysis of MRI data. Mean area (±SD) of low MR signal areas in aortic roots.
